# Hemobilia Secondary to Transjugular Intrahepatic Portosystemic Shunt Procedure: A Case Report

**DOI:** 10.3390/jcm1010015

**Published:** 2012-10-10

**Authors:** Dharmesh Kaswala, Divyang Gandhi, Andrew Moroianu, Jina Patel, Nitin Patel, David Klyde, Zamir Brelvi

**Affiliations:** Division of Gastroenterology and Hepatology, Department of Medicine, The University Hospital, New Jersey Medical School, University of Medicine and Dentistry of New Jersey (UMDNJ), 90 Bergen Street, DOC 2100, Newark, NJ 07103, USA; E-Mails: gandhidivyang@gmail.com (D.G.); amoroianu@yahoo.com (A.M.); chikujy@yahoo.com (J.P.); nitinmd@gmail.com (N.P.); klydeda@umdnj.edu (D.K.)

**Keywords:** hemobilia, hemetemesis, TIPS (transjugular intrahepatic portosystemic shunt), hepatic artery embolization

## Abstract

A 59 year-old woman with liver cirrhosis due to hepatitis C, complicated by refractory hepatic hydrothorax was treated with a TIPS (transjugular intrahepatic portosystemic shunt) procedure. The procedure was complicated by substantial gastrointestinal hemorrhage. EGD (esophagogastroduodenoscopy) was performed and revealed hemobilia. A hepatic angiogram was then performed revealing a fistulous tract between a branch of the hepatic artery and biliary tree. Bleeding was successfully stopped by embolization of the bleeding branch of the right hepatic artery. Hemobilia is a rare cause of upper gastrointestinal bleeding with an increasing incidence due to the widespread use of invasive hepatobiliary procedures. Hemobilia is an especially uncommon complication of TIPS procedures. We recommend that in cases of hemobilia after TIPS placement, a physician should immediately evaluate the bleeding to exclude an arterio-biliary fistula.

## 1. Introduction

The term hemobilia was first coined by Sandblom [1], when he described bleeding into the biliary tree following trauma. Hemobilia has now become widely recognized due to the improvements in diagnostic modalities and an increased index of clinical suspicion for the disorder. Hemobilia occurs when a fistula forms between a vessel of the splanchnic circulation (hepatic artery or portal vein) and the intrahepatic or extra-hepatic biliary system. Common causes include iatrogenic manipulation of the hepatobiliary system and trauma [2]. Management of hemobilia is aimed to stop bleeding, maintain continuous flow through the biliary system and treat the underlying etiology. Iatrogenic hemobilia after TIPS (transjugular intrahepatic portosystemic shunt) is extremely uncommon but several cases have been reported. [3,4,5]. We report iatrogenic hemobilia as a complication of TIPS procedure [6], which was successfully managed by transarterial embolization.

## 2. Case

**Figure 1:**
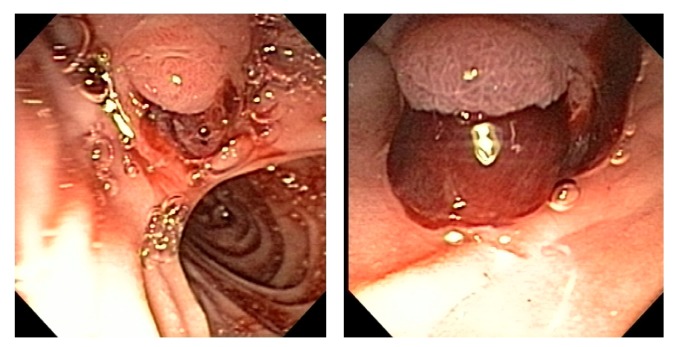
Ampulla of Vater showing hemobilia.

The patient is a 59 year-old female with history of liver cirrhosis due to hepatitis C, which was complicated by refractory ascites, hepatic hydrothorax, and hypertension, who visited to the hospital for shortness of breath and abdominal distension. She was found to have a right-sided pleural effusion. Pleural fluid analysis showed a serum ascites-albumin gradient (SAAG) >1.1, consistent with transudative effusion, most likely hepatic hydrothorax. A TIPS procedure was recommended for the treatment of the patient’s refractory hepatic hydrothorax. Pre-procedure: Total bilirubin 0.6 mg/dL, albumin 3.2 g/dL, ALP (alkaline phosphatase) 70 IU/L, ALT (alanine transaminase) 27 IU/L, AST (aspartate transaminase) 41 IU/L, INR (International Normalized Ratio) 1.5, hemoglobin 16.2 g/dL and serum creatinine 0.4 mg/dL. The TIPS procedure was successfully performed. After the procedure, the patient had multiple episodes of hemetemesis and her hemoglobin dropped to 8.9 g/dL. Packed red blood cell transfusion was administered and an EGD showed fresh, large blood clots emerging from the ampullary orifice consistent with hemobilia (Figure 1).

**Figure 2:**
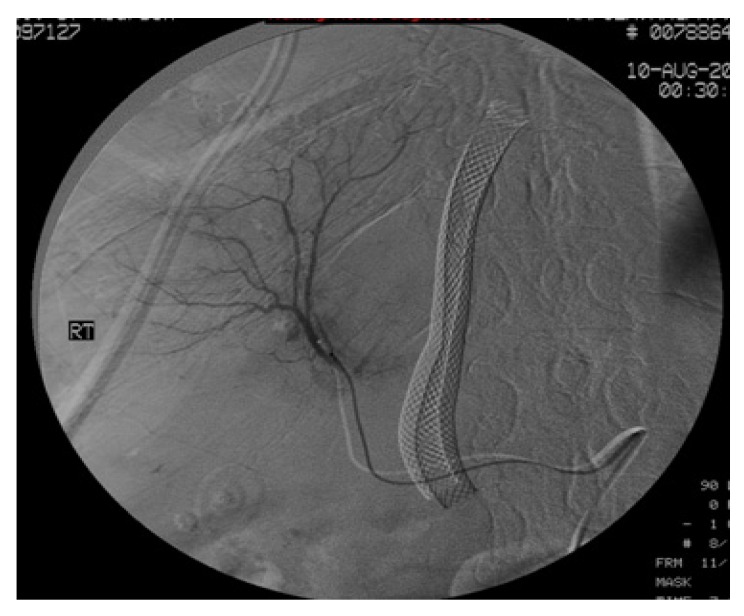
Sub-selective right hepatic arteriogram shows normal arborization of the selective artery injected. Shunt is in place.

**Figure 3:**
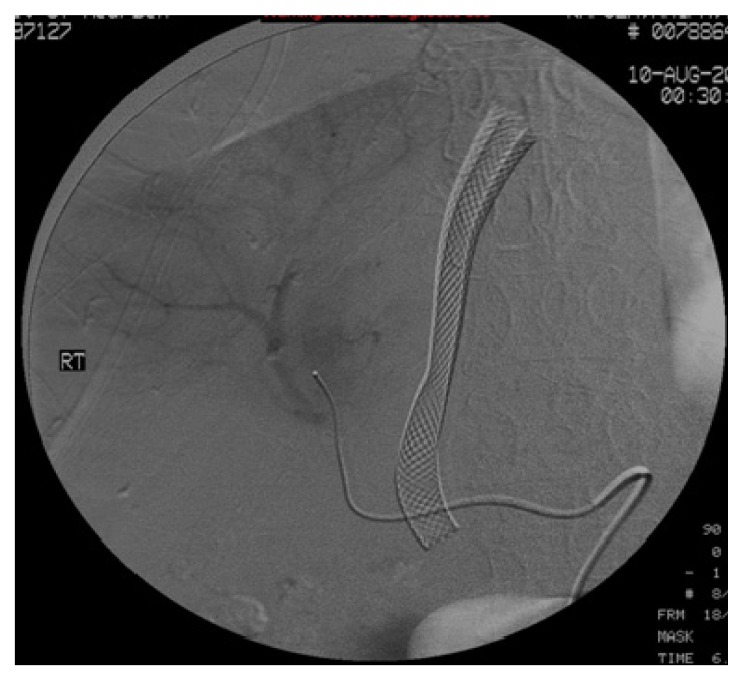
Delayed image of the arteriogram demonstrating the opacification of biliary tree, which indicates a fistula between hepatic artery and biliary tree.

She was immediately prepped for a hepatic angiogram with possible embolization. When the selected right hepatic artery that opacified the biliary tree was identified, a slurry of gelfoam and contrast was injected until there was a cessation of blood flow and resolution of the opacification of the biliary tree. See Figure 2 and Figure 3.

## 3. Discussion

Transjugular intrahepatic portosystemic shunt (TIPS) has been utilized in the treatment of portal hypertensive complications for more than 20 years. Indications for TIPS determined by controlled trials include management of variceal bleeding, refractory cirrhotic ascites, hepatorenal syndrome, gastric antral vascular ectasia, Budd Chiari syndrome, and refractory hepatic hydrothorax [7].

Table 1 shows the reported complications of tips [8].

**Table 1:** Reported complications of TIPS (transjugular intrahepatic portosystemic shunt)[8].

Complication of TIPS	Incidence
Direct procedure related mortality	0–2%
30 days mortality	7%–45%
Aggravated or new encephalopathy	5%–35%
Shunt stenosis/Occlusion	<5%
Infection (Infective endocarditis )	
Bleeding from capsular perforation	<5%
Extra hepatic puncture of portal vein	<5%
Parenchymal injury to biliary tree or hepatic artery	<5%
Stent related complications—migration, infection	<5%
Contrast induced renal failure	
Cardiac arrhythmias/Heart failure	<5%
Shunt related complications = Encephalopathy, liver failure, pulmonary hypertension	
Umbilical hernia	
Radiation injury to Skin	
Other possible complications Include—Fever, muscle stiffness, bruising on the neck at point of catheter insertion	

Iatrogenic hemobilia [9] may occur as a result of percutaneous liver procedures, liver or biliary operations, or therapeutic anticoagulation. Given the close proximity of bile duct radicals to the branches of the hepatic artery and portal vein, the substantial incidence of concurrent injury to these structures and fistula formation is not unexpected [10,11]. A 3.8% incidence of hepatic vascular abnormalities was found following percutaneous transhepatic cholangiography [12], a 5.4% incidence of hepatic vascular abnormalities following percutaneous liver biopsy, and a 26.2% incidence following the placement of indwelling transhepatic drainage catheters [13]. The frequency of clinical hemobilia ranges from less than 1% for liver biopsy [14] to 4% for transhepatic cholangiography, 3% to 14% for percutaneous transhepatic catheter drainage and <5% post-TIPS procedure. Our patient had end stage liver disease (ESLD). The TIPS procedure was done as a palliative therapy. In our case, a covered stent was not used. Furthermore, a survival benefit has not been demonstrated in covered stent. Therefore, an uncovered stent was used in our patient as she was not a transplant candidate and stent longevity was not of concern given the palliative nature of the procedure. 

The clinical presentation of hemobilia includes the Quinke triad: biliary colic, jaundice, and gastrointestinal bleeding, which may range from occult to massive bleeding. The initial diagnosis can be made with endoscopy, bleeding scan, or angiogram. Angiography with possible embolization is the treatment of choice for most cases of hemobilia because it can be both diagnostic and therapeutic [15,16]. Although only 12% of cases are initially diagnosed with endoscopy, it may confirm the diagnosis in an additional 30% of patients and help exclude other causes of upper gastrointestinal tract bleeding [17]. Biliary endoscopic procedures in current practice are helpful in the management of hemobilia [18]. Other diagnostic methods include computed tomography and ultrasonography [19]. If all of these measures fail to provide a diagnosis, or if the patient presents under emergency circumstances such as hemodynamic instability, the surgeon may be forced to do an exploratory laparotomy without a precise preoperative diagnosis.

The goals of therapy in cases of hemobilia are to stop the bleeding and to restore bile flow past any clot formation. Modalities used to stop bleeding include angiography with embolization, surgical intervention, and endoscopic electrocoagulation or photocoagulation. In the past, successful embolization of intrahepatic bleeding sites was affected by the technical inability to have selective arterial access. This lead to complications caused by nonselective embolization. However, today, angiography [15] is clearly the most efficacious method for controlling intrahepatic bleeding sources, with success rates above 95%. A complication of embolization includes hepatobiliary necrosis (6%), abscess formation (9%), bleeding (6%), and gallbladder fibrosis (2%).

It is interesting to note that hemobilia from a portal venous source, though exceedingly rare, is more likely to require surgical treatment [20]. Finally, surgical therapy should be considered as the treatment of choice when the cause of hemobilia constitutes an independent indication for such treatment, such as cases associated with cholelithiasis, cholecystitis, or resectable neoplasm.

The least commonly used option for managing hemobilia is that of expectant observation. Spontaneous cessation of bleeding occurs most often in patients who undergo percutaneous cholangiography or liver biopsy; therefore, this group merits observation as the primary management. Some authors have proposed the prophylactic administration of clot promoters such as absorbable gelatin sponges (Gelfoam, Upjohn, Kalamazoo, MI, USA) into percutaneous puncture tracts during withdrawal of the instruments or drains from the liver to reduces bleeding complication [21]. Endoscopic techniques for controlling hemorrhage and managing clots include nasobiliary drainage, sphincterotomy, and laser photocoagulation using small endoscopes placed through a catheter tract via access to the biliary tree [22]. These methods have only been reported anecdotally and will probably continue to have a role in selected cases. The complications of hemobilia are uncommon and include pancreatitis, cholecystitis, and cholangitis.

## 4. Conclusion

Physicians should be aware of hemobilia as one of the possible complications of TIPS and should be experienced in its management. Liver parenchymal puncture during a TIPS procedure may damage vascular structures such as the hepatic artery, portal vein, as well as bile duct. Therefore, we recommend that in cases of gastrointestinal hemorrhage after TIPS placement, a suspicion of hemobilia should be high on the differential diagnosis. After the diagnosis of hemobilia is confirmed by an upper endoscopy, hepatic angiogram should be done to visualize arterio-venous-biliary fistula. Today, transarterial embolization is the gold standard in the management of hemobilia. 
